# Using community-engaged methods to develop a study protocol for a cost analysis of a multi-site patient navigation intervention for breast cancer care

**DOI:** 10.1186/s12913-022-08192-y

**Published:** 2022-07-08

**Authors:** Serena Rajabiun, Victoria Xiao, Sharon Bak, Charlotte Robbins, Nicole Casanova, Howard J. Cabral, Stephenie C. Lemon, Jennifer S. Haas, Karen M. Freund, Tracy Battaglia, TRIP Consortium, Ted A. James, Ellen Ohrenberger, Jo Ellen Ross, Leo Magrini, Susan T. Gershman, Mark Kennedy, Anne Levine, Erica T. Warner, Cheryl R. Clark, William G. Adams, Sharon Bak, Tracy A. Battaglia, Janice Debrito, Katie Finn, Christine Gunn, Jackeline Hernandez, Naomi Y. Ko, Ariel Maschke, Katelyn Mullikin, Charlotte Robbins, Christopher W. Shanahan, Victoria Xiao, Howard J. Cabral, Clara Chen, Carolyn Finney, Christine Lloyd-Travaglini, Magnolia Contreras, Stephanie Loo, Rachel A. Freedman, Karen Burns White, Anne Marie Murphy, Beverly Moy, Jennifer S. Haas, Caylin Marotta, Mira Oravcova-Mejia, Sanja Percac-Lima, Amy J. Wint, Karen M. Freund, William F. Harvey, Amy M. LeClair, Susan K. Parsons, Dolma Tsering, Feng Qing Wang, Serena Rajabiun, Stephenie C. Lemon

**Affiliations:** 1 Department of Public Health, University of Massachusetts, Lowell, MA USA; 2 Boston University School of Medicine, Boston, MA USA; 3 Boston Medical Center, Boston, MA USA; 4 Department of Biostatistics, Boston University School of Public Health, Boston, MA USA; 5 University of Massachusetts Medical School, Worcester, MA USA; 6 Massachusetts General Hospital, Boston, MA USA; 7 Tufts Medical Center, Boston, MA USA

**Keywords:** Cost, Patient navigation, Breast cancer

## Abstract

**Background:**

Patient navigation is an evidence-based intervention for reducing delays in oncology care among underserved populations. In order to address the financial sustainability of this intervention, information is needed on the cost of implementing patient navigation in diverse healthcare settings. Because patient navigation programs and care settings are highly variable, this paucity of cost data creates difficulties in identifying best practices and decisions about the feasibility of implementing navigation programs within a health care system. One barrier to collecting these cost data is the lack of assessment tools available to support patient navigation programs. These tools must be relevant to the wide variety of navigation activities that exist in health care settings, and be flexible enough to collect cost data important to stakeholders in fee-for-service and value-based care environments.

**Methods and results:**

We present a novel approach and methods for assessing the cost of a patient navigation program implemented across six hospital systems to enhance timely entry and uptake of breast cancer care and treatment. These methods and tools were developed in partnership with breast oncology patient navigators and supervisors using principles of stakeholder engagement, with the goal of increasing usability and feasibility in the field.

**Conclusions:**

This methodology can be used to strengthen cost analysis and assessment tools for other navigation programs for improving care and treatment for patients with chronic conditions.

**Trial registration:**

NCT03514433

**Supplementary Information:**

The online version contains supplementary material available at 10.1186/s12913-022-08192-y.

## Background

Patient navigation is an evidence-based intervention for improving patient care and reducing disparities in cancer care and other chronic conditions [1]. Patient navigation entails individualized assistance offered to patients, families, and caregivers to help overcome healthcare system barriers and facilitate timely access to quality health and psychosocial care. Since its inception, navigation has been intended as an equity-based service tailored to community-specific needs and directed toward patients experiencing structural barriers related to care access with the ultimate aim of promoting equity.

However, there exists a paucity of economic evaluations for cancer patient navigation programs, and even fewer published methods for estimating the cost of a program [2–4]. The lack of data and tools for cost estimation creates challenges in making decisions for implementing, expanding, or improving navigation programs. With most navigation programs supported through time-limited grant funding mechanisms, sustainability is an ongoing challenge. Cost analysis provides information that can support health care providers and payers on decisions for integrating navigation programs into service delivery as a sustainable part of the health care workforce.

Previous economic evaluations of patient navigation programs in breast cancer care indicate that patient navigation programs are mixed in terms of cost-effectiveness [3–5]. Variation in these findings are attributed to intervention goals (i.e. time to diagnosis after an abnormal finding vs. initiation of treatment), time frame for analysis, and heterogeneity in methodology of cost metrics [5, 6]. Having reliable, valid cost metrics are critical to assess the economic impact and compare navigation programs [6].

Current economic evaluations of navigation programs in cancer care focus on a single navigation program and use navigators with various backgrounds and roles. Thus cost estimations may vary and making recommendations for replication and reimbursement can be challenging [7, 8]. In addition, few studies address the cost or cost-effectiveness of patient navigation across the cancer care continuum and are limited within a treatment modality, therefore missing the care coordination work across modalities that navigators perform. Another challenge is that most current studies examine cost using gross-costing estimates based on utilization and salaries and do not delineate specific roles and tasks, thus making it challenging to understand the potential value for navigation as part of the team [2–4, 9, 10].

Micro-costing is a direct cost estimation approach used in care coordination and patient navigation interventions for other chronic conditions, such as HIV [11–13]. Micro-costing involves the identification of resource use by staff activity logs or surveys and accounts for all relevant resources including provider time, supplies, equipment and facility space [11, 12]. The micro-costing approach assesses patient navigation programs by prospectively measuring the average cost of the intervention and allows for inter-site variations in cost. The strength of this approach is that it improves the reliability and validity of the cost estimate of the intervention by identifying key measures such as provider time, supplies, equipment and facility space that may not be standardized across settings. The variation in time spent per patient encounter makes other cost estimations, using only retrospective activity-based or gross-costing approaches based on utilization or financial data, too burdensome or inaccurate to obtain from hospital or health care systems, or too general to inform health care systems on costs associated with an intervention [13, 14].

Because navigation is intended to respond to a local community’s needs, navigation programs may vary depending on the level of patient acuity and specific program goals. To ensure that our cost-assessment methods aligned with the tasks and day-to-day activities conducted by navigators, we engaged breast oncology navigators and supervisors in the design process of our study. We selected this approach to allow a more pragmatic representation of the various activities that navigators contribute to patient outcomes in the health care delivery system. This approach builds off of principles of practice-based research, which aims to improve clinical research generalizability in real-life practices [15]. It also provides information that can help with sustainability by defining and standardizing the specific activities to be reimbursed by third party payors. This is often a challenge for non-clinical staff such as navigators in our health care delivery system [5].

In this article, we present our approach for the development of a cost analysis to assess a city-wide, multi-site patient navigation program to reduce disparities in breast cancer care in the greater Boston area. We describe our participatory methodology in developing and testing the feasibility of tools for micro-costing patient navigation programs. Using similar methods of community engagement, these tools can be tailored to navigation programs across the cancer care continuum and other chronic disease conditions.

## Methods

### Parent study

Translating Research into Practice (TRIP) is a city-wide patient navigation intervention spanning breast cancer treatment at six Boston area hospitals. TRIP is a community-engaged, cluster-randomized, stepped-wedge Type 1 hybrid effectiveness-implementation study that combines patient navigation, a systematic screening and referral system for social determinants of health (SDoH) via the Aunt Bertha social network platform (now called FindHelp), and a shared patient registry using the HIPAA-compliant REDCap platform in an integrated model of care to address disparities in breast cancer outcomes [7, 8]. TRIP comprises a subset of the navigation services offered at each of our clinical sites, as it is implemented within existing navigation programs but offers an enhanced standard of care to patients most likely to experience delays in care. TRIP navigation activities include an 11 step protocol focused on identifying and screening patients at risk for experiencing delays in care, conducting a social needs assessment and making referrals for service needs, following up with patients who miss appointments or are lost to care, and communicating with the health care team about patient needs and services. Details of the study intervention have been published elsewhere [8]. A key study goal is the success of intervention implementation in real world clinical settings across six implementation outcomes from the Proctor model: acceptability, local adoption, penetration, fidelity to the intervention protocol, sustainability, and cost of the intervention across each site [16, 17]. The methods described in this paper were developed to assess the cost of TRIP.

Using community-engaged methods, we developed a protocol and two tools for assessing TRIP costs:

1. A time-motion survey to collect variable labor costs of navigators and their supervisors, and
2. An administrative record worksheet to collect fixed labor costs, fixed non-labor costs, and variable non-labor costs including supplies and equipment.

All human subjects research was conducted in accordance to the guidelines of the Boston University/Boston University Medical Center (BUMC/IRB) Institutional Review Board. The study was approved and granted a waiver of informed consent by BUMC IRB (protocol H-37314).

### Community engagement

The community-engaged processes used to develop these cost tools include stakeholder engagement in the survey and protocol design process, an iterative process incorporating stakeholder feedback, and pilot testing with stakeholders [18, 19]. TRIP’s organizational structure includes the following stakeholder groups who co-created the cost methods and approaches with the investigator team:

1. A Clinical Advisory Panel (CAP) comprised of oncology providers and navigators from each of the six clinical sites that meets monthly and guides the implementation strategies, facilitates local adoption, and analyzes clinical outcomes;
2. An administrative core study team responsible for the overall study management including training, communications and data management and administrative support for the overall study; and
3. A network of TRIP breast oncology navigators and program supervisors at the six clinical sites who meet quarterly to discuss the local adoption of the protocol and share strategies [8].

In addition to these TRIP stakeholders, we reviewed the tools with navigators from the Women’s Health Network (WHN), a breast oncology program focusing on screening located at one of the clinical sites. The WHN is part of the state-funded Breast and Cervical Cancer Detection Program based at Boston Medical Center focusing on connecting women at risk for poor outcomes to breast and cervical screenings, with similar patient outreach and data tracking protocols.

The administrative core study team started by identifying existing micro-costing tools used in a patient navigation intervention for individuals with HIV [20] and a literature review of previous published cost studies on cancer screening [21, 22]. We began by adapting existing surveys and administrative record worksheets from prior studies and tailoring survey items using the 11 steps of the TRIP navigation protocol [8], with the goal of having mutually exclusive items and capturing all navigation activities conducted on behalf of TRIP patients.

The surveys were iteratively reviewed with TRIP and WHN supervisors and navigators, incorporating their feedback in between iterations. In response to feedback, the research staff created a survey key and protocol or instructions for ease of survey administration and standardization across the study sites and to ensure data quality. The CAP confirmed acceptability of the data collection protocol, including survey frequency and duration within each site’s clinic workflow.

For the administrative record worksheets, we adapted worksheets from a previous patient navigation intervention and tailored it to TRIP components, based on research staff experience with designing and implementing the intervention. The spreadsheet was then pilot tested with navigation program staff to ensure that the cost elements were applicable and reflective of resource utilization to implement the TRIP program in their setting.

## Results

### Feedback from pilot testing the data collection instruments

Navigators identified “making referrals [to outside social organizations to address identified social needs]” and “communication with third parties or outside organizations” as overlapping survey items that could lead to time being reported twice in the survey. Additionally, “referrals” were used by research staff to describe referrals to outside social organizations to address identified social needs, but navigators pointed out that this word could be confused with referrals to other care providers instead of referrals to social resources. Navigators also pointed out that “rescheduling a patient’s missed appointment” and “attempted patient contact and follow-up” could overlap, as their main mechanism of rescheduling a missed appointment was to call the patient. Finally, navigators asked for guidance on classifying waiting time, pointing out that because they frequently have to find the right time in a patient’s schedule to interact with them, they may have to wait for a patient to finish with the provider or for an interpreter to arrive.

Additionally, in the first round of testing the survey with WHN navigators, navigators expressed discomfort that the survey could be misconstrued as a performance review. Navigators also wanted to justify why an individual patient’s care may take longer – for instance, if a patient needs interpretation services, the navigator will spend time waiting for the interpreter. As such, the research team and navigators agreed that a survey introduction to set expectations for the navigator could address this discomfort and encourage navigators to accurately report the variation in time.

Navigators and supervisors also provided feedback on the survey protocol. For instance, the WHN supervisor pointed out that because navigators and supervisors are embedded in the clinical workflow, their time can be variable and limited. She suggested that research staff include the survey, survey key, and survey intro when setting up the introductory call by email so the navigators and supervisors can review it beforehand to expedite the call and to accommodate the flexible nature of their job. Below we summarize the final changes to the protocol and each data collection tool as a result of the pilot test.

#### Time-motion survey

We developed a time-motion survey that captures variable time spent on specific activities that can then be associated with variable labor costs. The navigator survey (Table 1) covers administrative tasks such as documentation and identifying eligible patients; direct patient encounters; patient care coordination such as communicating with the care team, other navigators, or third party organizations; and trainings and meetings. The supervisor survey (Table 2) covers administrative tasks, direct patient encounters, patient care coordination, supervision of navigators, and trainings and meetings.

**Table 1:** Navigator Survey. *Instructions:* Please think about your work each day with TRIP patients. A list of your current TRIP patients will be included with this survey. To the best of your knowledge please enter the amount of time (*in 15 minute increments,* i.e. *15, 30, 60, 90,* etc.) you spend on the following activities per day. Enter “0” if you did not perform that activity. Please round up in your estimates to the nearest 15 minutes

	Day									
Activities	1	2	3	4	5	6	7	8	9	10
**Date (mm/dd/yyyy)**										
**Administrative tasks**	**Time (minutes)**									
**1. Identifying eligible patients** (e.g. reviewing pathology reports or appointments)										
**2. Documenting navigation activities into data systems** (i.e. troubleshooting or entering information in REDCap, Excel, EHR, Aunt Bertha, etc.)										
**2a. Documenting in REDCap** (Of the time you listed in **#2**, how much of that time do you spend in REDCap entering information or troubleshooting?)										
**2b. Documenting in Aunt Bertha** (Of the time you listed in **#2**, how much of that time do you spend in Aunt Bertha entering information or troubleshooting?)										
**3. Viewing reports to monitor caseload** (e.g. viewing population-level reports in REDCap such as Initial Patient Search or Patient Tracking Report; Excel, etc.)										
**Direct patient contact**	**Time (minutes)**									
**4. Intake** (e.g. speaking with patient in-person or by phone to get information for TRIP intake form)										
**5. Social needs assessments** (e.g. conducting social needs assessment with patient)										
**6. Making referrals to address social needs** (e.g. giving patient list of referrals, helping patients apply for resources together)										
**7. Following up on social needs referrals** (e.g. following up with patient on incomplete/pending referrals for identified needs, asking patients on follow-up encounters if referrals were accessible and if needs were met)										
**8. (Re) scheduling clinical appointments** (e.g. coordinating patient appointments)										
**9. Patient education and support** (e.g. patient education around cancer care and resources)										
**Navigating on behalf of patient without direct patient contact**	**Time (minutes)**									
**10. Communication with care team** (e.g. communicating with healthcare team to discuss patient social needs, navigation services, and navigator concerns)										
**11. Communication with 3rd parties or outside organizations** (e.g. contacting insurances, contacting resources, arranging transport, making non-clinical appointments)										
**12. Communication with other navigators** (e.g. messaging other navigators to see if patient has been seen elsewhere, coordinating patients who are seeking 2nd opinions, warm hand-off of transfer patients)										
**13. Attempted patient contact and follow-up** (e.g. calling patient after missed appointment, searching registry to see if patient seen elsewhere, reaching out to patient’s support contacts, conducting home visit, sending letters)										
**Other TRIP-related tasks**	**Time (minutes)**									
**14. Training/Education** (e.g. attending training meetings or webinars. Includes travel time to and from off-site trainings and meetings.)										
**15. Cost Survey** (time spent filling out this cost survey)										
**16. Other**										
**# of Patients Navigated**	**Number of TRIP patients**									
**17. Number of TRIP patients navigated** (include whether you worked with that patient directly, or did navigation work on their behalf)	**n=**	**n=**	**n=**	**n=**	**n=**	**n=**	**n=**	**n=**	**n=**	**n=**

**Table 2:** Supervisor Survey. *Instructions:* Please think about your work in the past week related to TRIP or TRIP patients. A list of your current TRIP patients will be included with this survey. To the best of your knowledge please enter the amount of time (*in 15 minute increments,* i.e. *15, 30, 60, 90,* etc.) you spend on the following activities per day. Enter “0” if you did not perform that activity. Please round up your estimates to the nearest 15 minutes

	Day									
Activities	1	2	3	4	5	6	7	8	9	10
**Date (mm/dd/yyyy)**										
**Administrative tasks**	**Time (Minutes)**									
**1. Documentation on behalf of TRIP patients**										
**Direct patient contact or navigation**	**Time (Minutes)**									
**2. Direct navigation of TRIP patients**										
**Navigating on behalf of patient without direct patient contact**	**Time (Minutes)**									
**3. Communication related to TRIP patients**										
**TRIP supervision activities**	**Time (Minutes)**									
**4. Patient enrollment** (e.g. identifying TRIP patients)										
**5. Administrative Supervision** (e.g. managing navigator time, reviewing navigator workflow, preparing navigator feedback)										
**6. Clinical Supervision** (e.g. meeting with navigators to discuss patient cases, clinic-related activities)										
**7. Quality Assurance** (e.g. monitoring data in Aunt Bertha/REDCap/Excel trackers/EHR)										
**8. Trainings** (including travel time to and from off-site trainings)										
**9. Meetings** (including travel time to and from off-site meetings)										
**10. Cost Survey** (time spent filling out this cost survey)										
**11. Other**										
**# of Patients Navigated**	**Number of TRIP patients**									
**12. Number of TRIP patients navigated** (include whether you worked with that patient directly, or did navigation work on their behalf)	**n=**	**n=**	**n=**	**n=**	**n=**	**n=**	**n=**	**n=**	**n=**	**n=**

Each survey is accompanied by a key (Supplemental Tables 1a-b) to delineate the categories in which navigation or supervisory activities were conducted, especially in cases where time spent could be counted in multiple categories and overestimating time spent. For the navigator survey, these categories include:

- **Patient management activities**: Checking in about appointments, meetings, case notes and documentation and data entry for SDOH screener;
- **Direct patient encounters and work on behalf of the patient:** Accompaniment to medical visits, counseling/education about treatment, insurance related calls; meeting with community providers about resources; calls with physicians and patients about treatment plan;
- **Travel time:** Travel to training opportunities*;*
- **Training;** and
- **Supervision**

For supervisors, the survey categories includes:

- **Project administration:** TRIP related meetings;
- **Patient enrollment:** Identifying TRIP eligible patients;
- **Administrative supervision:** Time managing navigator case load and work hours;
- **Clinical supervision:** Patient case conferencing and treatment plans; team communication and coordination;
- **Quality assurance:** Monitoring SDoH data and patient registry;
- **Travel for meetings**; and
- **Training activities**

Navigators and supervisors tracked the number of hours spent on activities per day for TRIP patients over a period of 10 consecutive work days. (Tables 1 and 2).

To administer the survey and based on feedback from the pilot test, researchers performed the following steps:

1. **Introductory email:** The administrative core member emailed the navigator or supervisor regarding the goal of the survey, including the survey intro, survey, and key, and setting a 15-minute discussion to introduce the survey.
2. **Conducting an introductory meeting:** Using the survey introduction, the administrative core member introduced the cost survey protocol and expectations, survey, and survey key. The administrative core team member emphasized that the survey is not a performance review. The administrative core team member and navigator or supervisor confirmed the personnel’s working schedule to clarify planned dates of survey collection. Additionally, the administrative team member and navigator/supervisor confirmed the plan for frequency of check-in reminders, data collection method (paper or online format of survey), and timeframe for upload of survey data (generally once a week and at least twice during the survey period).
3. **Regular check-ins:** During the period of the survey collection, the administrative core team regularly checked the navigator or supervisor’s understanding of survey items and answered technical questions, based on the frequency of check-ins established at the introductory meeting. After the first study data upload, the administrative core team member checked the data and clarified any protocol or survey items with the navigator or supervisor. At the end of the survey, the administrative core team member checked data, clarified any protocol or survey items, and thanked the navigator or supervisor for their participation.

#### Survey and worksheet usage

During deployment of the tool to assess TRIP navigators, despite the variation in site characteristics in expected patient volume (three-fold difference) and navigator scope of role (nurse vs. lay navigator), they were able to complete the survey with minimal technical questions around transmitting the data to study staff. To date, the survey has been deployed at 5 sites, representing 7 navigators and 3 supervisors. All 7 navigators completed the survey with 100% completeness. Two of the supervisors completed the survey with 100% completeness, and 1 supervisor completed 3 of 10 days in the survey period before being lost to follow-up. The worksheet has been deployed at 5 sites and was filled out with 100% completeness.

#### Administrative data worksheet

The administrative record worksheet (Supplemental Tables 2a-d) captures fixed labor costs, fixed non-labor costs, and variable non-labor costs for both the administrative core team and staff at the six implementation sites. The worksheet captures three core components for costs associated with TRIP implementation:

1. **Startup costs** for the Administrative Core and Clinical Advisory Panel (CAP);
2. **Intervention implementation costs** for each of the six sites; and
3. **Maintenance costs** for the Administrative Core team.

Startup costs include personnel costs, meeting time for design of the social determinants of health (SDoH) screening tool and registry, technical support for the development of the patient registry, and training for the SDoH screener across the six site navigators and supervisors. Implementation costs for the sites include labor costs for the navigators and key supervision personnel, fringe benefits, supplies equipment and overhead costs that include facility, space and other related costs from the hospital setting. Maintenance costs for the administrative core team include personnel costs, supplies and equipment for administrative support for CAP and navigator meetings, and on-going data management and training activities.

To complete this worksheet, site administrative/financial managers for the TRIP study will gather cost elements from expenditure data for the grant using administrative reports during a specific fiscal time period. Given the nature of the stepped wedge study design, data is collected based on each site’s start up and implementation periods. Table 3 highlights the data collection periods for the time motion survey and administrative data worksheet.

**Table 3:** Data Collection Periods

Site type	Site #	Time motion survey time period	Cost worksheet intervention phase	Cost worksheet time period
Administrative	N/A	N/A	Start Up	9/1/2017–8/31/2018
			Maintenance	6/1/2019–5/31/2020
Clinical	1	6/1/2019–5/31/2020	Start Up	Hours trained prior to 9/17/2018
			Intervention	6/1/2019–5/31/2020
	2	6/1/2019–5/31/2020	Start Up	Hours trained prior to 12/17/2018
			Intervention	6/1/2019–5/31/2020
	3	6/1/2020–5/31/2021	Start Up	Hours trained prior to 3/18/2019
			Intervention	6/1/2020–5/21/2021
	4	6/1/2020–5/31/2021	Start Up	Hours trained prior to 6/20/2019
			Intervention	6/1/2020–5/21/2021
	5	6/1/2020–5/31/2021	Start Up	Hours trained prior to 11/25/2019
			Intervention	6/1/2020–5/21/2021

#### Analysis plan

We will conduct an economic analysis of the navigation versus usual care using a provider perspective. Our primary analysis will be to calculate the costs of implementing the intervention, including costs associated with developing and implementing the registry and SDOH screening systems, training and materials costs and navigator costs. The results of the survey and administrative records will be combined with chart abstraction for the primary clinical outcome of time to first treatment and for patient disease progression and number of ER visits and hospitalizations. There are our five cost measures: total costs, cost per patient served, average cost per site, cost per protocol activity, and cost per additional patient engaged in primary breast cancer treatment*.* Cost per protocol activity includes administrative tasks, direct patient contact, and coordination with providers (see Supplemental Tables 1 & 2). This cost will be calculated from supervisor and navigator survey on the estimated time spent on activities multiplied by the wage and fringe rates paid at each health care system. Finally, cost per additional patient engaged in primary breast cancer treatment will be calculated as dividing the total costs of the TRIP intervention by the additional patients engaged in TRIP versus the control group.

We will perform sensitivity analyses to estimate the range of intervention costs after varying inputs, including staff position (nurse vs lay navigator) or time spent per patient. Costs will be broken out by intervention component such that sites that already have core navigation functions can estimate the added costs for the registry and screen.

Our secondary focus will be to estimate the cost per outcome based on our primary outcome of time to receipt of first cancer treatment. We will plan to adjust outcomes based on disease severity as measured by patients’ disease staging and number of emergency room visits and hospital admissions. The analysis will only consider within the study period and therefore will not include discounting of future costs or effects. We will standardize all costs to 2019 US dollars. This approach will not assess cost effectiveness as no comparable control cost data exists to assess incremental costs.

## Discussion

The TRIP micro-costing study contributes to economic evaluations of navigation programs, specifically for assessing a city-wide approach to reducing disparities for breast cancer care. Our study is unique in using community-engaged methods to optimize relevance and quality of data on time spent and types of activities that contribute to navigator practice as part of an integrated care team. This type of information can be useful for developing standards and guidelines for reimbursement for health care providers and payers. The data also provide information to administrators and leaders for improving the quality and delivery of services to patients who may be high utilizers of services. The investment in connecting patients earlier to care through navigation programs can reduce the potential high cost of treatment at later stages of disease.

There are a number of strengths in our approach. Engaging navigator and supervisor feedback in the tool design process increased survey item match to the navigator’s job activities. Even after tailoring the survey items using the navigation protocol, navigators and supervisors provided feedback on items that were missing or redundant. Modifying the survey in response to navigator and supervisor feedback thus reduces the likelihood of misclassification bias of activities performed and improving the scope of activities performed by navigators as part of a healthcare team.

This stakeholder tool development approach reflects the community-engaged nature of the TRIP study [8]. Just as the overall TRIP protocol was developed in partnership with clinical stakeholders, the survey tool was developed in partnership with navigators and stakeholders. This process was feasible and accomplished over three 1-hr meetings (one with TRIP navigators, one with WHN navigators, and one with WHN supervisor). The data collection process was also reviewed and approved by the clinical advisory panel at a regularly scheduled meeting.

Our approach demonstrates that community-engaged tool development creates a data collection system that is tailored to the navigation protocol and can be used across variable sites. These tools have been deployed to assess costs of a navigation program spanning 5 hospitals. At 3 of 5 sites, there was a navigator supervisor, while at 2 of 5 sites, the navigators worked independently. One of the sites employed nurse navigators, while the other 4 sites employed lay navigators.

There are limitations to our community-engaged micro-costing approach. As they are tailored to the needs of the local patient community, the scope and nature of patient navigation programs is inherently variable. Therefore, no one cost estimation tool can be generalizable to all navigation programs in all health care settings. Our cost analysis includes a time-motion survey for variable labor costs of navigators and their supervisors, and gathering administrative records for fixed labor costs, fixed non-labor costs, and variable non-labor costs. The community-engagement method was tailored to a city-wide navigation program for breast cancer care in academic medical centers in Boston.

Second, the navigators and supervisors are not restricted to serve only TRIP patients and the data collected in the time motion survey reflects a specific point in time of TRIP navigation activities. Thus there could be potential missing or under/over estimation of time and activities documented in the survey. However, the feedback from the community-engagement process and development of the tool and protocol was designed to minimize this potential bias to improve data quality. Also, the community engagement approach utilized pre-existing relationships to enhance buy-in to the micro-costing approach with the clinical sites. The administrative core staff had pre-existing working relationships with the TRIP and WHN supervisor and navigators. Using a community-engagement approach may be harder if new relationships must first be formed across a city wide or regional network. Additionally, more members of the TRIP supervisor and navigator group from the sites could have been engaged.

We believe this community-engagement process is flexible and powerful by providing a cost assessment tool for patient navigation programs that can be customized to the specific navigation context. The outcomes from this cost measurement tool may be useful across different health systems, as evidenced by data collection from multiple clinical sites with different navigation program characteristics. The cost outcomes may be useful in both fee-for-service and value-based care systems, and especially for program managers to describe a position’s tasks, estimate the cost of navigation for budgets, and to make the business case for navigation in health systems. These types of tools are necessary to support sustainability of oncology navigation programs nationwide.

## Supplementary Information

**Additional file 1: Table S1a.** Navigator and Supervisor Survey Keys. **Table S1b.** Survey Key (Survey of Supervisor Time on TRIP Activities). **Tables S2a-d.** Cost Record Worksheets.

## Data Availability

The datasets generated and/or analysed during the current study are not publicly available since this was pilot data to develop and refine the study protocol but are available from the corresponding author on reasonable request.
