# Supplementary material for: Using community-engaged methods to develop a study protocol for a cost analysis of a multi-site patient navigation intervention for breast cancer care
Source: BMC Health Serv Res. 2022 Jul 8;22:881. doi: 10.1186/s12913-022-08192-y (PMC9264587; doi:10.1186/s12913-022-08192-y)
Supplement: Supplementary file 1 — Additional file 1: Table S1a. Navigator and Supervisor Survey Keys. Table S1b. Survey Key (Survey of Supervisor Time on TRIP Activities). Tables S2a-d. Cost Record Worksheets. [file 12913_2022_8192_MOESM1_ESM.docx]

**Supplemental Table 1a: Navigator and Supervisor Survey Keys**

**Survey Key (Survey of Navigator Time on TRIP Activities)**

| *This is a list of example tasks for each navigation activity listed on the Survey of Navigator Time on TRIP activities.*  *Feel free to use this survey key as a tool to aid in filling in the survey. Please fill in time spent on each activity in the survey.* | |
| --- | --- |
| **Navigation Activity** | **Description/Examples** |
| **ADMINISTRATIVE TASKS** | *Administrative tasks involved in TRIP navigation protocol such as identifying patients from reports, documenting navigation activities, or monitoring navigation caseload.* |
| **1. Identifying eligible patients** | - Reviewing pathology reports - Reviewing upcoming appointments - Reviewing patient information in EHR to check eligibility criteria |
| **2. Documenting navigation activities into data systems** | - Entering information in REDCap Registry forms (e.g. Intake, Encounter, Repeat Social Needs Screening, PN Transition/Completion forms) - Entering information in Excel trackers - Entering information in electronic health systems (e.g. Epic, etc.) - Entering information in Aunt Bertha (e.g. social needs screening results, referrals made, goals, etc.) - Trouble-shooting data systems (e.g. REDCap Registry, Excel, electronic health systems, Aunt Bertha) alone or with TRIP study team |
| **2a. Documenting in REDCap** | - Of the time you listed in **#2 (Documenting activities into data systems)**, how much of that time do you spend entering information in REDCap or troubleshooting REDCap? |
| **2b. Documenting in Aunt Bertha** | - Of the time you listed in **#2 (Documenting activities into data systems)**, how much of that time do you spend entering information in Aunt Bertha or troubleshooting REDCap? |
| **3. Viewing reports to monitor caseload** | - Viewing population-level reports in REDCap (e.g. Initial Patient Search, Patient Tracking Report) - Viewing population-level reports or trackers in Excel - Viewing other caseload management reports or trackers to monitor TRIP patient caseload and navigation activities |
| **DIRECT PATIENT CONTACT** | *Time spent directly interacting with patients as part of navigating patients using the TRIP protocol. Most likely involves interacting with patient in-person or over the phone.* |
| **4. Intake** | - Speaking with patient in-person or by phone to get information for TRIP intake form - Waiting to talk to patient for intake |
| **5. Social needs assessments** | - Speaking with patient in-person or by phone to administer standardized social needs assessment questions - Waiting to talk to patient to administer social needs assessment |
| **6. Making referrals to address social needs** | - Looking through list of resources with patient - Giving patient list of referrals to social resources to address identified social needs - Helping patients apply for social resources together (e.g. calling a resource together, filling out an application together) |
| **7. Following up on social needs referrals** | - Following up with patient by phone call or in-person regarding incomplete/pending referrals for identified needs - Asking patients on follow-up in-person visits or phone calls if past referrals were accessible and if identified needs were met |
| **8. (Re)scheduling clinical appointments** | - Coordinating/scheduling patient appointments with patient and providers - Rescheduling appointments for patients who missed an appointment |
| **9. Patient education and support** | - Patient education around cancer care and social resources - Emotional support around navigating cancer diagnosis/treatment or identified social needs |
| **navigating on behalf of patient without direct patient contact** | *Time spent conducting activities on behalf of a patient with the goal of coordinating their navigation and care. Does not involve directly interacting with patient by phone or in-person.* |
| **10. Communication with care team** | - Communicating with healthcare team in-person, by phone, or via electronic communication (e.g. EHR) to discuss patient social needs, navigation services, and navigator concerns. Healthcare team includes any member of the care team in your hospital who directly participates with providing the patient’s clinical care. This includes clinicians, in-house social workers and resource specialists, and administrative assistants for scheduling patient appointments. - Waiting to talk to member of care team |
| **11. Communication with 3^rd^ parties or outside organizations** | - Communicating with 3^rd^ party or outside organizations for coordinating patient services. These are parties who are not directly involved in patient care but may be involved in addressing patient social needs. Potential parties include insurances, social resources (e.g. food pantries, transportation services). Making an appointment on behalf of a patient at a social resource organization would fall in this category. |
| **12. Communication with other navigators** | - Contacting other TRIP navigators to see if a patient who cannot be contacted/is missing appointments has been seen at other hospitals. Contact can be by phone, REDCap Messenger, or other HIPAA-compliant method. - Coordinating a TRIP patient’s care with other TRIP navigators for patients who are receiving care at multiple institutions, seeking second opinions, or looking to transfer care. Contact can be by phone, REDCap Messenger, or other HIPAA-compliant method. - Contacting other TRIP navigators to coordinate care hand-off for a patient who transfer care between TRIP institutions. Contact can be by phone, REDCap Messenger, or other HIPAA-compliant method. |
| **13. Attempted patient contact and follow-up** | - Calling patient to attempt to reach patient after patient has missed appointment.   - If you reach the patient, please count the time conversing with the patient under the appropriate activity in Direct Patient Contact. - Searching Registry to see if patient has been seen at other TRIP sites.   - Count time conversing with other navigators under 12. Communication with other navigators. - Reaching out to patient’s support contacts - Conducting home visits (including travel time) - Sending letters to patient residence |
| **other TRIP-related tasks** | *Time spent on TRIP-related tasks, such as meetings, trainings, and trouble-shooting tools used for TRIP navigation.* |
| **14. Training/Education** | - Attending TRIP training meetings, TRIP navigator network meetings, or TRIP webinars. Includes travel time to and from off-site trainings and meetings. |
| **15. Cost Survey** | - Time spent filling out cost survey |
| **16-20. Other** | - Other TRIP navigation activities with TRIP patients that have not been listed above. List one activity per item and the time spent on each activity. |
| **# of TRIP Patients Navigated (number of individuals)** |  |
| **21. Number of TRIP patients navigated** | - Includes TRIP patients you worked with directly that day (e.g. interacted with them in-person or by phone) or did navigation work on their behalf (e.g. communicated with others on behalf of the patient, attempted to reach patient, etc.) |

**Supplemental Table 1b: Survey Key (Survey of Supervisor Time on TRIP Activities)**

| *This is a list of example tasks for each navigation activity listed on the Survey of Supervisor Time on TRIP activities.*  *Feel free to use this survey key as a tool to aid in filling in the survey. Please fill in time spent on each activity in the survey.* | |
| --- | --- |
| **Navigation Activity** | **Description/Examples** |
| **ADMINISTRATIVE TASKS** | *Administrative tasks involved in TRIP navigation protocol such as identifying patients from reports, documenting navigation activities, or monitoring navigation caseload.* |
| **1. Documentation on behalf of TRIP patients** | - Entering information in REDCap Registry forms (e.g. Intake, Encounter, Repeat Social Needs Screening, PN Transition/Completion forms) - Entering information in Excel trackers - Entering information in electronic health systems (e.g. Epic, etc.) - Entering information in Aunt Bertha (e.g. social needs screening results, referrals made, goals, etc.) - Viewing population-level reports in REDCap (e.g. Initial Patient Search, Patient Tracking Report) - Viewing population-level reports or trackers in Excel Viewing other caseload management reports or trackers to monitor TRIP patient caseload and navigation activities |
| **DIRECT PATIENT CONTACT** | *Time spent directly interacting with patients as part of navigating patients using the TRIP protocol. Most likely involves interacting with patient in-person or over the phone.* |
| **2. Direct navigation of TRIP patients** | - Speaking with patient in-person to get information for TRIP intake form - Speaking with patient by phone to get information for TRIP intake form - Speaking with patient in-person to administer standardized social needs assessment questions - Speaking with patient by phone to administer standardized social needs assessment questions - Looking through list of resources with patient - Giving patient list of referrals to social resources to address identified social needs - Helping patients apply for social resources together (e.g. calling a resource together, filling out an application together) - Following up with patient by phone call or in-person regarding incomplete/pending referrals for identified needs - Asking patients on follow-up in-person visits or phone calls if past referrals were accessible and if identified needs were met - Coordinating/scheduling patient appointments with patient and providers - Rescheduling appointments for patients who missed an appointment - Patient education around cancer care and social resources - Emotional support around navigating cancer diagnosis/treatment or identified social needs |
| **navigating on behalf of patient without direct patient contact** | *Time spent conducting activities on behalf of a patient with the goal of coordinating their navigation and care. Does not involve directly interacting with patient by phone or in-person.* |
| **3. Communication related to TRIP patients** | - Communicating with healthcare team in-person, by phone, or via electronic communication (e.g. EHR) to discuss patient social needs, navigation services, and navigator concerns. Healthcare team includes any member of the care team in your hospital who directly participates with providing the patient’s clinical care. This includes clinicians, in-house social workers and resource specialists, and administrative assistants for scheduling patient appointments. - Communicating with 3^rd^ party or outside organizations for coordinating patient services. These are parties who are not directly involved in patient care but may be involved in addressing patient social needs. Potential parties include insurances, social resources (e.g. food pantries, transportation services). Making an appointment on behalf of a patient at a social resource organization would fall in this category. - Contacting other TRIP navigators to see if a patient who cannot be contacted or is missing appointments has been seen at other hospitals. Contact can be by phone, REDCap Messenger, or other HIPAA-compliant method. - Coordinating a TRIP patient’s care with other TRIP navigators for patients who are receiving care at multiple institutions, seeking second opinions, or looking to transfer care. Contact can be by phone, REDCap Messenger, or other HIPAA-compliant method. - Contacting other TRIP navigators to coordinate care hand-off for a patient who transfer care between TRIP institutions. Contact can be by phone, REDCap Messenger, or other HIPAA-compliant method. - Calling patient to attempt to reach patient after patient has missed appointment. If you reach the patient, please count the time conversing with the patient under 2. Direct navigation of TRIP patients. - Searching Registry to see if patient has been seen at other TRIP sites. - Reaching out to patient’s support contacts - Conducting home visits (including travel time) - Sending letters to patient residence |
| **TRIP Supervision activities** | *Time spent on TRIP-related supervision tasks, such as meetings, trainings, and trouble-shooting tools used for TRIP navigation.* |
| **4. Patient Enrollment** | - Reviewing pathology reports to identify TRIP patients - Reviewing upcoming appointments to identify TRIP patients - Reviewing patient information in EHR to check eligibility criteria |
| **5. Administrative Supervision** | - Managing navigator time via meetings, trackers, reports, etc. - Reviewing navigator workflows - Preparing feedback for navigators |
| **6. Clinical Supervision** | - Meeting with navigators to discuss patient cases or clinic-related activities |
| **7. Quality Assurance** | - Monitoring data in Aunt Bertha or REDCap registry to ensure data completeness and accuracy |
| **8. Trainings** | - Attending TRIP trainings (in-person or webinar). Includes travel time to and from off-site trainings. |
| **9. Meetings** | - Attending TRIP meetings (in-person or webinar). Includes travel time to and from off-site meetings. |
| **10. Cost survey** | - Time spent filling out this cost survey |
| **11-15. Other** | - Other TRIP supervisor activities that have not been listed above. List an individual activity in each row and the time spent on that activity. |

**Supplemental Tables 2a-d: Cost Record Worksheets**

| **Supplemental Table 2a: Start Up Costs for Administrative Core** | | |
| --- | --- | --- |
| **Administrative core site** |  | |
| **Time period of data collection** |  | |
| **Cost Item** | **details** | **amount** |
| **Personnel** |  |  |
| Program Coordinator (%FTE spent on implementation x salary) |  |  |
| Research Staff (%FTE spent on implementation strategies x salary) |  |  |
| Data analyst (%FTE spent on platform building x salary) |  |  |
| Other supervision support time |  |  |
| Fringe rate (if not included) |  |  |
| **Technical Platforms** |  |  |
| REDCap (Registry platform) |  |  |
| Aunt Bertha (SDoH screen + referrals platform) |  |  |
| **Training costs** |  |  |
| a. Personnel costs (if not accounted for above) |  |  |
| b. Materials/supplies |  |  |
| c. Travel |  |  |
| d. Other direct costs (please describe in notes) |  |  |
| **Other materials/supplies** |  |  |
| **Subtotal of direct costs** |  |  |
| **Overhead/indirect rate** |  |  |
| **TOTAL COST PER SITE** |  | |
| **Notes** |  | |

**Supplemental Tables 2a-d: Cost Record Worksheets**

| **Table 2b: Maintenance Costs for Administrative Core** | | |
| --- | --- | --- |
| **Administrative core site** |  | |
| **Time period of data collection** |  | |
| **Cost Item** | **details** | **amount** |
| **Personnel** |  |  |
| Program Coordinator (%FTE spent on implementation x salary) |  |  |
| Research Staff (%FTE spent on implementation strategies x salary) |  |  |
| Data analyst (%FTE spent on platform building x salary) |  |  |
| Other supervision support time |  |  |
| Fringe rate (if not included) |  |  |
| **Technical Platforms** |  |  |
| REDCap (Registry platform) |  |  |
| Aunt Bertha (SDoH screen + referrals platform) |  |  |
| **Training costs** |  |  |
| a. Personnel costs (if not accounted for above) |  |  |
| b. Materials/supplies |  |  |
| c. Travel |  |  |
| d. Other direct costs (please describe in notes) |  |  |
| **Other materials/supplies** |  |  |
| **Subtotal of direct costs** |  |  |
| **Overhead/indirect rate** |  |  |
| **TOTAL COST PER SITE** |  | |
| **Notes** |  | |

**Supplemental Tables 2a-d: Cost Record Worksheets**

| **Supplemental Table 2c: Start Up Costs for Clinical Work** | | |
| --- | --- | --- |
| **Clinical site** |  | |
| **Time period of data collection** | **Prior to (Go Live date)** | |
| **Cost Item** | **details** | **amount** |
| **Personnel** |  |  |
| Patient Navigators (hourly wage / % FTE/salary) |  |  |
| Supervisor (hourly wage / % FTE/salary) |  |  |
| Clinical champion (% FTE spent on TRIP (e.g. CAP calls, site visits) x salary or hourly wage) |  |  |
| Fringe rate (if not included) |  |  |
| **Training costs** |  |  |
| a. Personnel costs (if not accounted for above) |  |  |
| b. Materials/supplies |  |  |
| **Other materials/supplies** |  |  |
| **Subtotal of direct costs** |  |  |
| **Overhead/indirect rate** |  |  |
| **TOTAL COST PER SITE** |  | |
| **Notes** |  | |

| **Supplemental Table 2d: Implementation Costs for Clinical Work** | | |
| --- | --- | --- |
| **Clinical site** |  | |
| **Time period of data collection:** |  | |
| **Cost Item** | **details** | **amount** |
| **Personnel** |  |  |
| Patient Navigators (hourly wage / % FTE/salary) |  |  |
| Program Supervisor/Manager (hourly wage / % FTE/salary) |  |  |
| Clinical champion (% FTE spent on implementation (e.g. CAP calls, site visits) x salary or hourly wage) |  |  |
| Tech support (hourly wage / % FTE/salary) -- IT support, generating potential patient lists, etc. |  |  |
| Fringe benefits (if not included in salary) |  |  |
| **Other Direct costs** |  |  |
| **Subtotal of Direct Costs** |  |  |
| **Overhead/indirect rate (equipment)** |  |  |
| **TOTAL COST PER SITE** |  | |
| **Notes: *Other direct costs include*:** educational promotional materials site intervention staff use to recruit or part of patient services; Incentives/food/ gifts cards/transportation support for clients to come to appointments; Other materials for intervention or supplies. ***Overhead rates/indirect rates***: include expenses related to facility and administration such as space, equipment (computers), communications (phones). |  | |
